# CLONAL HEMATOPOIESIS IN A CENTENARIAN COHORT

**DOI:** 10.1093/geroni/igz038.394

**Published:** 2019-11-08

**Authors:** Aparna Bhutkar, Anastasia Gurinovich, Thomas T Perls, Paola Sebastiani, Stefano Monti

**Affiliations:** 1 Boston University, Boston, Massachusetts, United States; 2 Boston University School of Medicine, Boston, Massachusetts, United States; 3 Boston University, Department of Biostatistics, Boston, Massachusetts, United States

## Abstract

Mosaicism, the presence of two or more genotypically or karyotypically distinct populations of cells in a single individual, plays an important role in human disease. Mosaicism can result in mutations and/or chromosomal alterations such as loss, gain, or copy-number neutral loss of heterozygosity. Clonal mosaicism and its relationship to aging and cancer, has been previously studied, and earlier work suggests that clonal mosaicism tends to increase with age. The aim of our research is to use genotype data of centenarians to explore the relationship between extreme longevity and mosaic chromosomal alterations (mCAs). To this end, we analyzed genome-wide genotypes from blood-derived DNA of 338 individuals from the New England Centenarian Study. The participants in this dataset ranged from 45 to 112 years of age. For the detection of mCA events, we used MoChA (https://github.com/freeseek/mocha), a bcftools extension, that predicts mCAs based on B-allele frequency (BAF) and log2 intensity(R) ratio (LRR), and uses long-range phase information to increase sensitivity. Chromosomal alteration events, including whole chromosome events, were detected in 180 out of the 338 individuals. A total of 165 duplications, 97 deletions, and 9 copy-number neutral loss of heterozygosity were detected. Additionally, there were 42 events whose copy number state could not be determined with high confidence. 236 events out of the 313 were detected in individuals aged 100 and older. Our analysis of chromosomal alteration frequency by age indicates that, within centenarians, the proportion of individuals with mCAs significantly decreases with increased age (p < 0.05, correlation -0.73).

